# Correlation of Foraminal Parameters with Patient-Reported Outcomes in Patient with Degenerative Lumbar Foraminal Stenosis

**DOI:** 10.3390/jcm12020479

**Published:** 2023-01-06

**Authors:** Yu-Tsung Lin, Jun-Sing Wang, Wei-En Hsu, Yu-Hsien Lin, Yun-Che Wu, Kun-Hui Chen, Chien-Chou Pan, Cheng-Hung Lee

**Affiliations:** 1 Department of Orthopedics, Taichung Veterans General Hospital, Taichung 40705, Taiwan; 2 Department of Medicine, School of Medicine, National Yang Ming Chiao Tung University, Taipei 11221, Taiwan; 3 Division of Endocrinology and Metabolism, Department of Internal Medicine, Taichung Veterans General Hospital, Taichung 40705, Taiwan; 4 Ph.D. Program in Translational Medicine, National Chung Hsing University, Taichung 40227, Taiwan; 5 Department of Post-Baccalaureate Medicine, College of Medicine, National Chung Hsing University, Taichung 40227, Taiwan; 6 Department of Computer Science and Information Engineering, Providence University, Taichung 43301, Taiwan; 7 Department of Rehabilitation Science, Jenteh Junior College of Medicine, Nursing, and Management, Miaoli 35664, Taiwan; 8 Department of Food Science and Technology, Hung Kuang University, Taichung 43304, Taiwan

**Keywords:** lumbar foraminal stenosis, foraminal parameters, quantitative analysis, MRI, transforaminal lumbar interbody fusion, patient-reported outcome, superior foraminal width

## Abstract

The relationship between quantitative anatomic parameters in MRI and patient-reported outcomes (PROs) before and after surgery in degenerative lumbar foraminal stenosis remains unknown. We included 58 patients who underwent transforaminal lumbar interbody fusion (TLIF) for single-level degenerative disc disease with foraminal stenosis between February 2013 and June 2020. PROs were evaluated using the visual analog scale (VAS) for back and leg pain, Oswestry Disability Index (ODI), and EuroQol-5D (EQ-5D). The foraminal parameters assessed using preoperative MRI included foraminal height, posterior intervertebral disc height, superior and inferior foraminal width, and foraminal area. The correlation between foraminal parameters and PROs before operation, at 1 year follow-up, and change from baseline were assessed. The associations between the aforementioned parameters were examined using linear regression analysis. The analysis revealed that among these parameters, superior foraminal width was found to be significantly correlated with ODI and EQ-5D at the 1 year follow-up and with change in ODI and EQ-5D from baseline. The associations remained significant after adjustment for confounding factors including age, sex, body mass index, and duration of hospital stay. The results indicated that in degenerative lumbar foraminal stenosis, decreased superior foraminal width was associated with better improvement in disability and quality of life after TLIF.

## 1. Introduction

Lumbar foraminal stenosis is an important etiology of lumbar radiculopathy. According to the previous literature, the incidence rate of lumbar foraminal stenosis is approximately 8–11% [1]. Narrowing of the lumbar neuroforamen occurs with spondylosis. The loss of intervertebral disc height results in cephalad subluxation of the superior articular process of the inferior vertebra, which decreases the height and area of the foramen in the craniocaudal dimension. Further, facet joint osteophyte and hypertrophic ligamentum flavum also lead to a decrease in foraminal width and area in the anterior–posterior dimension [2]. These changes may lead to entrapment of the lumbar nerve and dorsal root ganglion and cause clinical symptoms. However, a considerable proportion (~50%) of patients respond poorly to conservative treatment [3], and in such cases, surgical intervention may be required.

Surgical management of foraminal stenosis involves decompressing the exiting nerve root with or without spinal fusion. Decompression without fusion has the potential risk of reduced disc height after surgery, which may result in recurrent stenosis for which revision surgery may be required [4]. Transforaminal lumbar interbody fusion (TLIF) with unilateral facetectomy can provide adequate decompression of the affected exiting nerve root and achieve circumferential fusion in a single approach, resulting in both radiographic and clinical improvements after surgery [5,6].

However, there are considerable variations in clinical response to surgical intervention in patients with lumbar foraminal stenosis [7,8]. Magnetic resonance imaging (MRI) is often used for preoperative assessment in this patient population. There are several MRI-based grading systems [9,10,11]; however, most of them are qualitative rather than quantitative, and the relationship between anatomic parameters and patient-reported outcomes (PROs) remains unknown. In this study, we sought to investigate the association between quantitative anatomic parameters (obtained using preoperative MRI) and PROs in patients undergoing TLIF for foraminal stenosis.

## 2. Materials and Methods

### 2.1. Study Population

This was a single-center, retrospective cohort study. We enrolled patients who underwent TLIF for single-level degenerative disc disease with foraminal stenosis and symptoms of radicular leg pain in the Department of Orthopedics at our hospital between February 2013 and June 2020. Patients who were admitted for revision and those who had concomitant segmental instability [12], multi-level spinal stenosis, central canal stenosis [13], or postoperative follow-up of <1 year were excluded from this study. Moreover, those with lumbosacral foraminal stenosis (L5-S1) were also excluded due to the significant morphology difference compared to other lumbar levels [2,14]. We measured several anatomic parameters using preoperative MRI. Various PROs were determined at baseline (preoperatively) and at post-operative follow-up, which were evaluated using the Visual Analog Scale (VAS) for back and leg pain, Oswestry Disability Index (ODI), and EuroQol-5D (EQ-5D). Post-operative evaluations were performed at 6 months and 1 year after operation. This study was conducted in accordance with the Declaration of Helsinki and approved by the Institutional Review Board of Taichung Veterans General Hospital (TVGH-CE21337A).

**Figure 1:**
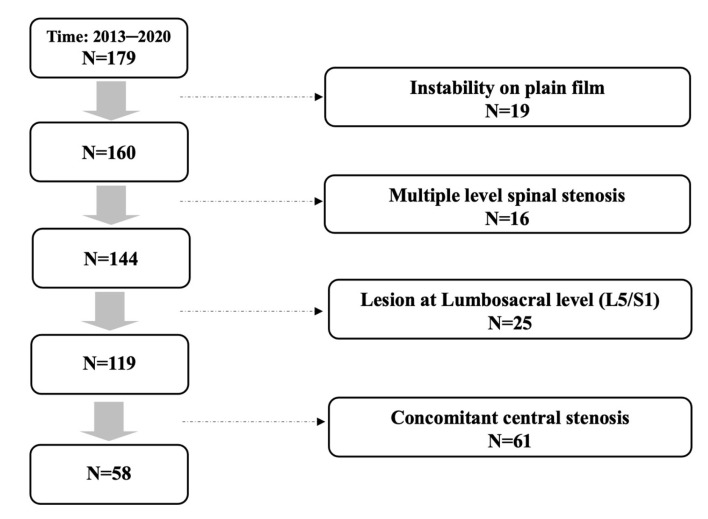
Patient inclusion flow diagram.

In total, 179 patients received primary single level TLIF for spinal stenosis in our institute. Among these patients, 19 with concomitant segmental instability on plain film were excluded, and 16 patients with multiple level stenosis, 25 patients with lesion at lumbosacral level, and 61 patients with concomitant central stenosis were ruled out after reviewing pre-operative MRI of every included individual. A total of 58 consecutive patients remained and were included in the study. The patient inclusion flow diagram is presented at Figure 1.

### 2.2. MRI Parameters and PRO Measurement

**Figure 2:**
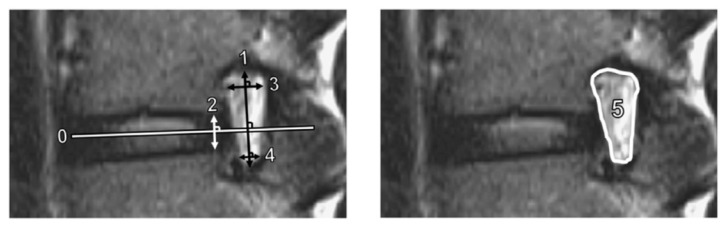
Measurements of magnetic resonance imaging parameters. Line 1 is the foraminal height; Line 2, disc height; Line 3, superior foraminal width; Line 4, inferior foraminal width; and Area 5, foraminal area. The anterior–posterior axis of the intervertebral disc (Line 0) was identified first. Lines 1 and 2 were constructed perpendicular to Line 0. Lines 3 and 4 were constructed parallel to Line 0.

We measured several quantitative foraminal parameters using preoperative lumbar spine MRI. Foraminal height was defined as the longest distance between the superior and inferior pedicles, while posterior intervertebral disc height was the shortest distance between the adjacent endplates at the posterior half of an intervertebral disc. In addition, superior and inferior foraminal widths were defined as the maximum width of the superior and inferior parts of the foramen, respectively [14]. The foraminal area was determined using the border of perineural fat on the magnetic resonance images [15]. Figure 2 shows the methods used to measure these parameters. To standardize the measurement of the parameters, the anterior–posterior axis of the intervertebral disc (Line 0) was first identified. Subsequently, the foraminal (Line 1) and disc (Line 2) heights were determined perpendicular to Line 0, while the superior foraminal (Line 3) and inferior foraminal (Line 4) widths were measured parallel to Line 0.

PROs were assessed at baseline (preoperatively) and at the 1-year follow-up; they included the VAS for pain in the back and leg, ODI, and EQ-5D. The VAS (scored from 0 to 10) was used to evaluate patients’ perceived pain intensity (0 = no pain), and the ODI (0–100) was used to assess patients’ functional status (0 = no disability). Further, the EQ-5D was used to assess patients’ quality of life, which was converted to an index score before analyses (a higher score denotes a better quality of life).

### 2.3. Statistic Analysis

PROs at baseline and at the 1-year follow-up postoperatively were compared using the Wilcoxon signed-rank test. Correlations between anatomic parameters and PROs at baseline and at the 1year follow-up and changes from baseline were assessed using Spearman’s rank correlation. Associations between the aforementioned parameters were examined using linear regression analysis after adjusting for age, sex, body mass index (BMI), and duration of hospital stay. All statistical analyses were performed using Statistical Package for the Social Sciences (IBM SPSS Statistics for Windows, version 22.0; International Business Machines Corp., Armonk, NY, USA), and the level of statistical significance was set at *p* < 0.05.

### 2.4. Surgical Procedure

Every included patient received surgery of conventional open TLIF. During the procedure, a midline skin incision was made, and the fascia was incised with paravertebral muscles elevated and dissected from the spine. Unilateral facetectomy at the symptom side of the level was performed to allow sufficient decompression of the exiting nerve root. Discectomy and endplate preparation were then performed through Kambin’s Triangle, followed by placement of interbody cage. Bilateral pedicle screw and rod were inserted, and correct screw and cage position was confirmed with intraoperative fluoroscopy.

The pedicle screw-rod instrumentation included: 46.5% CD Horizon Legacy (Medtronic Sofamor Danek, Minneapolis, MN, USA), 41.4% Wiltrom spinal fixation system (Wiltrom Co. Ltd., Hsinchu city, Taiwan), 8.6% Illico (Alphatec Spine, Carlsbad, CA, USA), 22.4% Xia (Stryker Spine, Allendale, NJ, USA) and 5.2% Sextant (Medtronic Sofamor Danek, Minneapolis, MN, USA). The interbody cages used included: 51.7% Capstone (Medtronic Sofamor Danek, Minneapolis, MN, USA), 34.5% TM-Ardis (Zimmer Biomet Spine, Westminster, CO, USA), and 13.8% SD (Alphatec Spine, Carlsbad, CA, USA).

## 3. Results

### 3.1. Patient Population Demographics

Fifty-eight patients were enrolled in the study. All patients underwent unilateral facetectomy and nerve root decompression, followed by single-level TLIF with standard bilateral posterior pedicle screw instrumentation. Table 1 shows the characteristics of the study population. Fifty-two of the 58 (89.7%) patients underwent surgery at the level of L4/L5. The median duration of hospital stay was 6.0 days (interquartile range, 5.0–7.0 days).

### 3.2. Clinical Improvement after Surgery

Table 2 shows the PRO scores at baseline and at the 1 year follow-up. The VAS scores for back and leg pain significantly improved after operation (both *p* < 0.001). The ODI decreased significantly, while the EQ-5D significantly increased at the 1-year follow-up (both *p* < 0.001).

### 3.3. Correlations between Anatomic Parameters and PROs

The correlations between anatomic parameters and PROs at baseline, at 6 months follow-up, at 1 year follow-up, and change from baseline are shown in Table 3. At baseline, none of the parameters obtained from MRI were correlated with any of the PRO measures. At the 6 months follow-up, the superior foraminal width was significantly correlated with the ODI (ρ = 0.344, *p* < 0.01) and EQ-5D (ρ = −0.277, *p* < 0.05) as well as with changes in the ODI (ρ = −0.49, *p* < 0.01) and EQ-5D (ρ = −0.43, *p* = 0.01) from baseline. At the 1-year follow-up, the superior foraminal width was significantly correlated with the ODI (ρ = 0.341, *p* = 0.010) and EQ-5D (ρ = −0.306, *p* = 0.022) as well as with changes in the ODI (ρ = 0.468, *p* < 0.001) and EQ-5D (ρ = −0.431, *p* = 0.001) from baseline.

**Figure 3:**
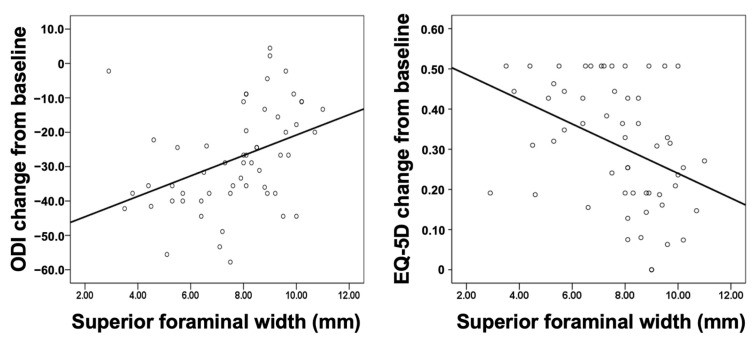
Scatter plot of the superior foraminal width against changes in the ODI (**left panel**) and EQ-5D (**right panel**) from baseline to the 1 year follow-up. EQ-5D, EuroQol-5D; ODI, Oswestry Disability Index.

Figure 3 shows the scatter plot of superior foraminal width against change in the ODI and EQ5D from baseline. The smaller the superior foraminal width, the greater the decrease in the ODI and improvement in the quality of life (EQ-5D).

The finding of linear regression also revealed the significant association between superior foraminal width and change in the ODI (β coefficient 2.971, 95% CI 1.059 to 4.884, *p* = 0.003) and EQ-5D (β coefficient −0.031, 95% CI −0.051 to −0.011, *p* = 0.003) from baseline at 1 year follow up (Table 4). These associations remained significant after adjusting for age, sex, BMI, and duration of hospital stay (*p* = 0.007 and 0.004 for ODI and EQ-5D, respectively). We also examined the associations in subgroups of sex and age in unadjusted models. The associations were more prominent in female patients and in those aged ≥65 years.

## 4. Discussion

In this study, we investigated the associations between anatomic parameters determined using preoperative MRI and PROs in patients who had undergone TLIF for lumbar foraminal stenosis. We demonstrated that the superior foraminal width was independently associated with changes in the ODI and EQ-5D from baseline to the 1-year follow-up. Considerable variations in improvements of PRO after surgical intervention for lumbar foraminal stenosis are not uncommon [7,8,16]. Our findings are clinically relevant, as we reported the association between anatomic parameters determined on the basis of preoperative MRI (commonly used for preoperative assessment) and improvements in important PROs for spine surgery after TLIF. To the best of our knowledge, this is the first study to investigate the correlation between quantitative anatomic parameters and patient-reported outcomes in patients with foraminal stenosis.

The factors associated with unsatisfactory outcomes after decompression surgery have been described previously [17,18]. However, factors associated with PROs after TLIF for foraminal stenosis remain unclear. There have been several qualitative MRI-based grading systems for lumbar foraminal stenosis. Wildermuth et al. introduced a grading system based on the degree of epidural fat obliteration [9]. Lee et al. developed a 4-point MRI grading system based on contact from different directions of the nerve root with surrounding structures, which provides nearly perfect inter- and intra-observer agreement [10]. Recently, Sartoretti et al. expanded the 4-point grading system to a more detailed 6-point grading system [11]. However, little is known regarding the clinical correlation of these classifications in the previous literature. Park et al. investigated the clinical correlation of the Wildermuth and Lee grading systems in a nonsurgical population [19]; however, the clinical symptoms in the study were evaluated by neurologic examination rather than based on objective clinical parameters such as PROs.

Findings on MRI have been shown to be associated with response to treatment and the possibility of failed conservative therapy in patients with foraminal stenosis. According to a study conducted by Farshad et al. [20], a T2 melting sign > 25% on MRI, which indicates a high degree stenosis, can predict VAS improvement > 50% after periradicular steroid infiltration. In another three-dimensional MRI study conducted by Yamada et al. [21], foramina occupied ≥ 50% by fat obliteration were likely to fail conservative treatment, with a positive predictive value of 75%. In our study, we found that in surgery-indicated foraminal stenosis, the severity of stenosis is also related to the improvement of functional status and quality of life after TLIF surgery.

Among all of the foraminal parameters, we found that superior foraminal width is the most clinically relevant parameter. Our finding may be anatomically reasonable. As the lumbar spinal nerve root and dorsal root ganglion run through the upper part of the foramen, the superior foraminal width might be an optimal parameter associated with the degree and risk of nerve root compression. The development of lumbar foraminal stenosis usually begins with a decrease in intervertebral disc height in degenerative disc disease [2], which results in subsequent loss of foraminal height. Besides, a protruded disc will further compress the inferior part of a neuroforamen. Therefore, a decrease in the foraminal height, disc height and inferior foraminal width may be observed in the early stages of the disease. However, a decrease in the superior foraminal width may not be observed until progression of disc degeneration when severe loss of intervertebral disc height and subluxation of superior articular process result in subsequent ligamentum flavum buckling. At this stage of the disease, the existing nerve root is, therefore, severely compressed between the superior articular facet and the posterior vertebral body at the superior part of the neuroforamen in the anteroposterior direction. Hence, it is reasonable to expect that a decrease in superior foraminal width before surgery, which possibly implies a more severely compressed nerve root, would be associated with better postoperative improvements in PROs (Figure 3, Table 4), as confirmed in this study.

There were no significant associations between the anatomic parameters obtained from preoperative MRI and the preoperative VAS scores for back and leg pain, ODI, and EQ-5D (Table 3). Sigmundsson et al. [22] reported a poor correlation between dural sac area and the walking distance, ODI, SF-36, EQ-5D, and levels of back and leg pain in patients with central spinal stenosis. In another cross-sectional study [23], only 17.5% of patients with severe central stenosis assessed by MRI were symptomatic. Kuittinen et al. found no relationships between the MRI findings and symptoms or walking capacity in patients with lateral lumbar spinal canal stenosis [24]. Similarly to the results of these previous reports, our results showed no significant associations between the MRI findings and clinical symptoms, functional status, and quality of life among patients selected for surgery. However, we found that the superior foraminal width was associated with ODI and EQ-5D changes from baseline 1 year after surgical intervention of TLIF. We observed that the associations were more prominent in female patients and in those aged ≥65 years (Table 4). Due to the small number of patients in each subgroup, these findings need to be confirmed in future studies.

This study has several limitations. First, due to the strict inclusion criteria we applied, the sample size of the present study was relatively small. However, concomitant segmental instability and central stenosis, which may be important coexisting pathologies in patient with foraminal stenosis, were strictly ruled out by individually reviewing pre-operative MRI images to limit bias. Second, patients with foraminal stenosis at the lumbosacral (L5/S1) level were excluded due to the anatomic difference compared to other lumbar levels [2]; thus, further study focused on this level may be required in the future. Finally, inter-observer variation during the measurement of MRI parameters may be a confounding factor in this study; however, given the strong inter-observer agreement (a kappa value > 0.8) in this study, we believe that inter-observer variation did not significantly affect our findings. Despite these limitations, this is the first study to investigate the correlation between foraminal anatomic parameters and patient-reported outcomes in patients who received TLIF for degenerative lumbar foraminal stenosis. Therefore, our findings may help surgeons in the future to identify patients who may demonstrate greater clinical improvement 1 year after TLIF.

## 5. Conclusions

Anatomic foraminal parameters assessed using preoperative MRI were independently correlated with patient-reported outcomes in degenerative lumbar foraminal stenosis. Decreased superior foraminal width was associated with better improvement in both functional status and quality of life after TLIF.

## Figures and Tables

**Table 1:** Characteristics of the study population.

Number of Patients	58
Age, years	64.0 (56.0, 68.3)
Female, *n* (%)	37 (63.8)
Body mass index, kg/m^2^	26.5 (23.5, 29.5)
Side of symptoms, *n* (%)	
Right	25 (43.1)
Left	31 (53.4)
Bilateral	2 (3.4)
Level of operation, *n* (%)	
L2/L3	3 (5.2)
L3/L4	3 (5.2)
L4/L5	52 (89.7)
Duration of hospital stay, days	6.0 (5.0, 7.0)
Parameters from MRI	
Foraminal height, mm	16.5 (14.5, 18.3)
Disc height, mm	5.5 (4.3, 6.4)
Superior foraminal width, mm	8.1 (6.4, 9.1)
Inferior foraminal width, mm	2.4 (0.0, 3.2)
Foraminal area, mm^2^	63.0 (48.8, 79.3)

Values are median (interquartile range) or *n* (%). MRI, magnetic resonance imaging.

**Table 2:** Parameters for symptoms and quality of life before operation and at 1 year follow-up.

	Before Operation	1 Year after Operation	*p* Value
VAS—back pain	7.0 (5.3, 8.0)	2.0 (0, 3.0)	<0.001
VAS—leg pain	7.0 (6.3, 8.8)	0 (0, 0)	<0.001
ODI	53.3 (44.4, 57.8)	20.0 (11.1, 37.2)	<0.001
EQ-5D	0.34 (0.34, 0.46)	0.77 (0.65, 0.85)	<0.001

Values are median (interquartile range). EQ-5D, EuroQol-5D. ODI, Oswestry Disability Index. VAS, Visual Analogue Scale.

**Table 3:** Correlations between parameters from MRI and patient-reported outcomes (PROs).

	VAS—Back Pain	VAS—Leg Pain	ODI	EQ-5D
	Before operation (baseline)			
Foraminal height	−0.015	−0.045	0.042	0.022
Disc height	0.097	−0.049	0.155	0.011
Superior foraminal width	−0.060	−0.062	−0.066	0.180
Inferior foraminal width	0.044	−0.089	−0.032	−0.068
Foraminal area	−0.002	0.039	−0.040	0.035
	6 months after operation			
Foraminal height	−0.089	−0.158	0.009	0.012
Disc height	−0.069	−0.118	−0.017	−0.063
Superior foraminal width	0.058	0.087	0.344 **	−0.277 *
Inferior foraminal width	0.021	0.025	0.136	−0.165
Foraminal area	0.117	0.053	0.109	−0.188
	1 year after operation			
Foraminal height	−0.013	−0.073	−0.001	0.069
Disc height	0.115	−0.087	0.022	−0.043
Superior foraminal width	0.238	0.239	0.341 *	−0.306 *
Inferior foraminal width	0.225	0.143	0.134	−0.163
Foraminal area	0.204	0.260	0.187	−0.234
	Change from baseline at 6 months (Δ PROs)			
Foraminal height	0.025	0.084	0.102	0.064
Disc height	0.179	0.018	0.104	−0.022
Superior foraminal width	−0.082	−0.124	−0.490 **	−0.430 **
Inferior foraminal width	−0.016	−0.173	−0.203	−0.086
Foraminal area	−0.067	−0.02	−0.241	−0.196
	Change from baseline at 1 year (Δ PROs)			
Foraminal height	0.012	−0.029	−0.096	0.087
Disc height	−0.021	0.025	−0.076	−0.020
Superior foraminal width	0.195	0.162	0.468 ***	−0.431 **
Inferior foraminal width	0.172	0.202	0.203	−0.086
Foraminal area	0.108	0.117	0.278 *	−0.254

Values are ρ for Spearman correlation. * *p* < 0.05, ** *p* < 0.01, *** *p* < 0.001. EQ-5D, EuroQol-5D. MRI, magnetic resonance imaging. ODI, Oswestry Disability Index. VAS, visual analog scale.

**Table 4:** Associations of superior foraminal width with change from baseline in ODI and EQ-5D.

	ODI Change from Baseline at 1 Year		EQ-5D Change from Baseline at 1 Year	
	β Coefficient (95% CI)	*p*	β Coefficient (95% CI)	*p*
Model 1	2.971 (1.059, 4.884)	0.003	−0.031 (−0.051, −0.011)	0.003
Model 2	2.343 (0.534, 4.151)	0.012	−0.026 (−0.045, −0.006)	0.011
Model 3	2.468 (0.715, 4.221)	0.007	−0.028 (−0.046, −0.009)	0.004
Male	1.727 (−1.885, 5.340)	0.329	−0.016 (−0.052, 0.019)	0.352
Female	4.004 (1.781, 6.226)	0.001	−0.043 (−0.067, −0.019)	0.001
<65 years	1.210 (−0.743, 3.163)	0.215	−0.019 (−0.048, 0.011)	0.203
≥65 years	4.035 (0.922, 7.149)	0.013	−0.037 (−0.063, −0.011)	0.007

Model 1, unadjusted. Model 2, adjusted for age and sex. Model 3, adjusted for variables in Model 2 plus body mass index and duration of hospital stay. EQ-5D, EuroQol-5D. ODI, Oswestry Disability Index.

## Data Availability

All data are available upon reasonable request from the corresponding author.

## References

[1] Jenis L.G., An H.S. (2000). Spine update: Lumbar foraminal stenosis. Spine.

[2] Orita S., Inage K., Eguchi Y., Kubota G., Aoki Y., Nakamura J., Matsuura Y., Furuya T., Koda M., Ohtori S. (2016). Lumbar foraminal stenosis, the hidden stenosis including at L5/S1. Eur. J. Orthop. Surg. Traumatol..

[3] Choi Y.K. (2019). Lumbar foraminal neuropathy: An update on non-surgical management. Korean J. Pain.

[4] Kim H.-J., Jeong J.-H., Cho H.-G., Chang B.-S., Lee C.-K., Yeom J.S. (2015). Comparative observational study of surgical outcomes of lumbar foraminal stenosis using minimally invasive microsurgical extraforaminal decompression alone versus posterior lumbar interbody fusion: A prospective cohort study. Eur. Spine J..

[5] Lowe T.G., Tahernia A.D., O’Brien M.F., Smith D.A. (2002). Unilateral transforaminal posterior lumbar interbody fusion (TLIF): Indications, technique, and 2-year results. Clin. Spine Surg..

[6] Fujibayashi S., Neo M., Takemoto M., Ota M., Nakamura T. (2010). Paraspinal-approach transforaminal lumbar interbody fusion for the treatment of lumbar foraminal stenosis. J. Neurosurg. Spine.

[7] Hallett A., Huntley J.S., Gibson J.N.A. (2007). Foraminal stenosis and single-level degenerative disc disease: A randomized controlled trial comparing decompression with decompression and instrumented fusion. Spine.

[8] Parlato C., Iavarone A., Gentile M., Albanese R., Moraci A. (2013). Outcome of lumbar intervertebral foraminal stenosis surgery and depression. Eur. Neurol..

[9] Wildermuth S., Zanetti M., Duewell S., Schmid M.R., Romanowski B., Benini A., Böni T., Hodler J. (1998). Lumbar spine: Quantitative and qualitative assessment of positional (upright flexion and extension) MR imaging and myelography. Radiology.

[10] Lee S., Lee J.W., Yeom J.S., Kim K.-J., Kim H.-J., Chung S.K., Kang H.S. (2010). A practical MRI grading system for lumbar foraminal stenosis. Am. J. Roentgenol..

[11] Sartoretti E., Wyss M., Alfieri A., Binkert C.A., Erne C., Sartoretti-Schefer S., Sartoretti T. (2021). Introduction and reproducibility of an updated practical grading system for lumbar foraminal stenosis based on high-resolution MR imaging. Sci. Rep..

[12] Gopinath P. (2015). Lumbar segmental instability: Points to ponder. J. Orthop..

[13] Steurer J., Roner S., Gnannt R., Hodler J. (2011). Quantitative radiologic criteria for the diagnosis of lumbar spinal stenosis: A systematic literature review. BMC Musculoskelet. Disord..

[14] Hasegawa T., An H.S., Haughton V.M., Nowicki B.H. (1995). Lumbar foraminal stenosis: Critical heights of the intervertebral discs and foramina. A cryomicrotome study in cadavera. J. Bone Jt. Surg..

[15] Ren Z., Liu A., Yang K., Wang D., Buser Z., Wang J.C. (2017). Evaluation of changes in lumbar neuroforaminal dimensions in symptomatic young adults using positional MRI. Eur. Spine J..

[16] Bakkai A., Wai E., Roffey D. (2013). Does the degree of foraminal stenosis affect the outcome of decompressive surgery in patients with lumbar spinal stenosis? Comparison of pain scores and disability at baseline and follow-up. Orthop. Proc..

[17] Chang S.-B., Lee S.-H., Ahn Y., Kim J.-M. (2006). Risk factor for unsatisfactory outcome after lumbar foraminal and far lateral microdecompression. Spine.

[18] Yamada K., Matsuda H., Nabeta M., Habunaga H., Suzuki A., Nakamura H. (2011). Clinical outcomes of microscopic decompression for degenerative lumbar foraminal stenosis: A comparison between patients with and without degenerative lumbar scoliosis. Eur. Spine J..

[19] Park H.J., Kim S.S., Lee S.Y., Park N.H., Rho M.H., Hong H.P., Kwag H.J., Kook S.H., Choi S.H. (2012). Clinical correlation of a new MR imaging method for assessing lumbar foraminal stenosis. Am. J. Neuroradiol..

[20] Farshad M., Sutter R., Hoch A. (2018). Severity of foraminal lumbar stenosis and the relation to clinical symptoms and response to periradicular infiltration—Introduction of the “melting sign”. Spine J..

[21] Yamada K., Abe Y., Satoh S., Yanagibashi Y., Hyakumachi T., Masuda T. (2017). A novel diagnostic parameter, foraminal stenotic ratio using three-dimensional magnetic resonance imaging, as a discriminator for surgery in symptomatic lumbar foraminal stenosis. Spine J..

[22] Sigmundsson F.G., Kang X.P., Jönsson B., Strömqvist B. (2011). Correlation between disability and MRI findings in lumbar spinal stenosis: A prospective study of 109 patients operated on by decompression. Acta Orthop..

[23] Ishimoto Y., Yoshimura N., Muraki S., Yamada H., Nagata K., Hashizume H., Takiguchi N., Minamide A., Oka H., Kawaguchi H. (2013). Associations between radiographic lumbar spinal stenosis and clinical symptoms in the general population: The Wakayama Spine Study. Osteoarthr. Cartil..

[24] Kuittinen P., Sipola P., Aalto T.J., Määttä S., Parviainen A., Saari T., Sinikallio S., Savolainen S., Turunen V., Kröger H. (2014). Correlation of lateral stenosis in MRI with symptoms, walking capacity and EMG findings in patients with surgically confirmed lateral lumbar spinal canal stenosis. BMC Musculoskelet. Disord..

